# Nanoencapsulation of *Achyrocline satureioides* (Lam) DC—Essential Oil and Controlled Release: Experiments and Modeling

**DOI:** 10.3390/pharmaceutics16121560

**Published:** 2024-12-05

**Authors:** Caroline G. F. da Silva, Rafaela R. Petró, Jéssica H. de Castro, Rafael N. Almeida, Eduardo Cassel, Rubem M. F. Vargas

**Affiliations:** Unit Operations Lab, School of Technology, Pontifical Catholic University of Rio Grande do Sul, Av. Ipiranga 6681-Prédio 30, Bloco F, Sala 208, Porto Alegre 90619-900, Brazil; caroline.finkler@gmail.com (C.G.F.d.S.); rafaela.petro@acad.pucrs.br (R.R.P.); rnolibos@gmail.com (R.N.A.); cassel@pucrs.br (E.C.)

**Keywords:** nanocapsules, nanoemulsion, polycaprolactone, air diffusion, perfumery radar

## Abstract

**Background/Objectives:** Degradation by physical and chemical agents affects the properties of essential oils; therefore, this study aimed to protect the volatile compounds present in essential oils through biopolymer encapsulation. **Methods:** The *Achyrocline satureioides* (Lam) DC. essential oil was obtained by steam distillation at 2.5 bar. The nano-sized physical coating of the active oil core resulted in an optimal polymer/oil ratio of 1:3 and particle diameter of 178 nm. The particle morphology was evaluated using scanning electron microscopy and transmission electron microscopy. The inclusion of the essential oil in the polymer was confirmed using thermogravimetric analysis. **Results:** The pH of the formulation remained stable for 90 days, and controlled release and encapsulation efficiencies were evaluated. Formulations were evaluated using the perfumery radar technique, which indicated a predominantly woody profile. The diffusion of fragrant compounds in the air was assessed over time and mathematically modeled. **Conclusions:** The produced nanostructures were efficient for the controlled release of volatile compounds from the essential oil of *Achyrocline satureioides*.

## 1. Introduction

*Achyrocline satureioides* (Lam) DC., popularly known as marcela, is a perennial shrub native to southeastern South America. Different extracts obtained from the aerial parts are traditionally used as digestive, anti-inflammatory, antispasmodic, antidiabetic, anti-asthmatic, emmenagogue, and antifebrile agents [1]. Its extracts are increasingly used in fragrances, carbonated drinks, alcoholic drinks, and flavorings for tobacco produced in Mercosur because of their deep and bitter taste [2,3]. Marcela essential oil is also used as an insect repellent [4,5] and exhibits antioxidant activity [6]. The aromatic profile of the *A. satureioides* essential oil was recently characterized by gas chromatography coupled with the olfactometry technique, presenting β-caryophyllene (31–26%), α-pinene (9–23%), α-copaene (6–9%), and α-humulene (4–7%) as major compounds. However, olfactometric analysis indicated that only α-pinene, α-terpineol, α-copaene, and β-caryophyllene are significant to olfactory perception [7].

High-volatility compounds (top notes) define the fragrance identity, which can easily disappear during production, storage, and use. Aroma encapsulation aims to mitigate these losses and improve the stability of aromatic products [8]. Many novel and promising encapsulation techniques have been reported for the encapsulation of aroma and flavor compounds, such as nano- and microemulsions [9]. Solid colloidal particles with nanometric-scale dimensions have been produced for various purposes [10], including antimicrobial activity [11] and aroma release [12]. In the perfumery sector, the release of aromas from different structures is a decisive step in the development of new perfumes and new aromatized materials [13]. Owing to their complexity, fragrances are qualitatively classified into olfactory families, referring to the predominant fragrant notes. *A. satureioides* extracts obtained by high-pressure extractions were classified as potential candidates for use in aroma development [14]. The perfumery radar technique was developed to classify perfumes into olfactory families using physicochemical models and qualitative descriptors [15]. However, the diffusion process from a point source is well described by a predictive and validated model for the axial and three-dimensional hypotheses [16,17]. By combining these two approaches, the availability of fragrant compounds at the liquid–vapor interface for different colloidal systems was evaluated in terms of their controlled release [18,19], which is important for the design and evaluation of new fragrant materials or products containing fragrances. 

This study investigated the nanoencapsulation of the *A. satureioides* essential oil by polycaprolactone, a biodegradable and biocompatible polymer which is extensively used in the development of new eco-friendly materials. The influence of both the encapsulating material and the encapsulated essential oil, as well as the essential oil nanoemulsion, on the properties of the nanostructures was analyzed. The nanoparticles produced and the nanoemulsions were characterized using dynamic light scattering (DLS), zeta potential, polydispersity index (PDI), field-emission scanning electron microscopy (FESEM), and transmission electron microscopy (TEM). Stability and release were assessed based on the control of pH and antioxidant activity over time. Additionally, the headspace concentrations of the nanoparticles, nanoemulsions, and pure *A. satureioides* essential oil were evaluated. 

## 2. Materials and Methods

### 2.1. Essential Oil

Plant materials were purchased from a supplier in Santo Antônio da Patrulha, Rio Grande do Sul, Brazil. Only the dry aerial parts of *A. satureioides*, inflorescences, and branches were used. The material was separated from the stems, conditioned in 700 g lots, and kept at room temperature in a dry place until extraction.

The essential oil was obtained from a pilot steam-distillation plant. The unit consists of a boiler with a capacity of 20 L (electric resistor of 2 kW), extraction vessel with a capacity of 10 L, heat exchanger, and liquid–liquid separator. Further details regarding this unit can be found in Souza Junior et al. [20]. The same units were used to obtain *A. satureioides* essential oil in previous studies [7]. The extractions were performed at 2.5 bar for 1 h and repeated until a satisfactory accumulated volume of oil was obtained for encapsulation tests. The mass of plant material used in all extractions was 700 g.

### 2.2. GC-MS Analysis

The essential oil samples were diluted in cyclohexane (Merck P.A.) at a ratio of 1:5 (*v*/*v*). The chemical composition of the essential oil was analyzed using a gas chromatograph (7890A), coupled to a mass spectrometer (5975C), both from Hewlett Packard–Agilent Technologies (Santa Clara, CA, USA). An HP-5MS column (30 m × 25 mm, 0.25 μm) was used with ultrapure helium as the carrier gas with 0.8 mL/min flow and an injector temperature of 250 °C. The analysis method started at 60 °C, which was maintained for 8 min, increased by 3 °C/min to 180 °C, held for 1 min, increased by 20 °C/min to 250 °C, and maintained here for 10 min. The interface temperature between the chromatograph and the MS was 230 °C, ionization voltage was 70 eV and mass range analyzed was 40 to 450 u. A 1:80 split was used with 1 μL injections. The compounds were identified by comparing their retention indices, determined from a series of alkanes (C_8_–C_20_), with those reported in the literature [21], and by comparing their mass spectra. All analyses were performed in triplicate.

### 2.3. Preparation of Nanoformulations

The nanoprecipitation technique proposed by Fessi et al. [22] with small modifications used poly-ε-caprolactone, PCL, (Purac Biomaterials, Amsterdam, The Netherlands), essential oil and 0.016 g of Span 60 (Merck, Darmstadt, Germany) as an organic phase that was diluted in 10 mL of propanone. This methodology has been widely used for the production of polymeric nanoparticles for different applications and has already been applied to *A. satureioides* oils for the treatment of mice infected with *Trypanosoma evansi* [23,24]. Five formulations were prepared, Mi with i = 1 to 5, associated with the following proportions between polymers and essential oil masses: 1:1, 1:2, 1:3, 1:4, and 1:5, respectively. The polymer mass used was always 25 mg. Also, 10 mL of a solution with mass fraction of 0.16% of Tween 80 in water was prepared. To produce the nanocapsules, the organic phase was slowly pipetted into the aqueous solution in a vortex with high rotation speed to promote maximum dispersion of the phases. Next, the propanone was removed using a rotary evaporator operating isothermally at 35 °C. A formulation without essential oil, with only the polymer, was prepared to be the blank in the antioxidant activity tests. The formulation for the nanoemulsions was the same as that used to produce the nanocapsules, only without the addition of the polymer (PCL). All samples were stored at 25 °C to await the assays, which were all performed in triplicate. 

### 2.4. Characterization of the Nanoformulations

The hydrodynamic diameter of the nanocapsules was assessed using dynamic light scattering (DLS). The determination of zeta potential and PDI was performed in a ZetaSizer^®^ Nanoseries (Malvern, England) instrument. Characterization was performed by field-emission scanning electron microscopy (FEG-SEM) TEM. FEG-SEM analysis was performed using Fei Inspect F50 equipment. One drop of each sample was applied to carbon tape and metalized for 80 s using gold sputtering equipment. For particle visualization, a working distance of 12 mm, radial force of 20 kV, and magnification of 2500–5000 times were used. TEM was performed using a Zeiss Axio Imager. For these analyses, 0.1 mL of the formulations were diluted in 1 mL of Milli-Q water, and uranyl acetate (2% *m*/*v*) was used as a contrast agent.

The reproducibility of the results was evaluated by calculating the coefficient of variation (CV), defined as the ratio of the standard deviation to the mean of the results obtained. All experiments were performed in triplicate. Aiming to identify significant differences between the mean values obtained, the Tukey test (*p* < 5) and analysis of variance (ANOVA) were used. All statistical tests were performed using tools available in MATLAB 14 (MATLAB and Statistics Toolbox Release 2014a, The MathWorks, Inc., Natick, MA, USA). 

### 2.5. Nanoencapsulation Efficiency

The essential oil mass lost during the process was evaluated because the solvent removal step occurs at reduced pressure, which may lead to the volatilization of terpenic compounds. First, aliquots of 1 mL were filtered in 0.22 μm PTFE filters to evaluate the free essential oil in the suspension (not encapsulated). The nanocapsule suspension, nanoemulsions, and filtrate were submitted to a liquid–liquid extraction with dichloromethane, and 1 μL of the organic phase was injected into the chromatograph to quantify the amount of essential oil present. A rapid method of analysis [25] started at 60 °C, was maintained for 4 min, increased at a rate of 20 °C/min up to 250 °C, and was then held for 2 min. Calibration curves using α-pinene (Sigma-Aldrich, St. Louis, IL, USA, ≥99%) and β-caryophyllene (Merck, ≥80%) were constructed to quantify the compounds. The samples were diluted in hexane, and a split ratio of 1:55 was used. All experiments were performed in triplicate.

The encapsulation efficiency, determined by Equation (1) [26], was calculated for the major compounds α-pinene and β-caryophyllene, since the low mass percentage of minor compounds could interfere in their quantification due to the high relative experimental error:

(1) $$EE\%=\frac{m_{if}-m_{uf}}{m_{i0}}*100$$

where $m_{i0}$ is the mass of the initially added compound, $m_{if}$ is the mass of the compound in the suspension of nanoparticles formed, and $m_{uf}$ is the mass of the compound present in the filtrate.

The nanocapsules were also subjected to thermogravimetric analysis (TGA) in an STD-Q600 instrument (TA Instruments, New Castle, DE, USA) to verify the presence of oils in the polymer nanocapsules. For this analysis, aliquots of 5 mL of the formulations were lyophilized and 1 mg of the resulting powder was heated from 25 °C to 800 °C at a heating rate of 10 °C/min and maintained at the final temperature for 3 min [27]. The 1 mg samples of the oils were subjected to the same analysis, and a lyophilized polymer (PP_lio_) was used as a reference.

### 2.6. Stability and Release

The pH and antioxidant activities of the different systems were monitored over time to determine their release and stability. The pH values of the nanocapsules and nanoemulsions were monitored for 2 and 4 months, respectively. pH measurements were performed using a Quimis pH meter, model Q400AS, calibrated in the acidic range.

The antioxidant activities of the nanoparticles and nanoemulsions were determined by the DPPH method [28], based on the capture carried out by the antioxidant agent of the DPPH radical (2,2-diphenyl-1-picryl-hydrazyl) (Sigma^®^) producing a decrease in absorbance of a 60 μM DPPH solution, changing its color from purple to light yellow. The absorbance was measured using a Biospectro SP-220 spectrophotometer at a wavelength of 515 nm [29] in glass cuvettes, and evaluated for a month. On the first day (day zero), the samples were evaluated 1 h and 2 h after the beginning of the reaction. Subsequently, the absorbance was measured every five days. In all readings, the absorbance of the 60 μM DPPH solution was measured as a blank. The DPPH scavenging percentage *(*%SR) was calculated using Equation (2):

(2) $$\%SR=\left[\left(\frac{{ABS}_{control}-{ABS}_{sample}}{{ABS}_{control}}\right)\right]*100$$

where ${ABS}_{sample}$ is the sample absorbance of the nanoparticle or nanoemulsion and ${ABS}_{control}$ refers to the absorbance of the control formulation (blank); the absorbances of the PP formulation and pure DPPH are the controls for the nanoparticles and nanoemulsions, respectively.

### 2.7. Perfumery Radar

The gas phase in equilibrium with the essential oil, nanocapsules, and nanoemulsions was analyzed using static headspace chromatography. Samples (1 mL) of each formulation were left to rest for 24 h in a 15 mL vial to reach phase equilibria. Gas-phase aliquots of 100 μL were sampled with a gastight syringe and analyzed by gas chromatography with the same equipment and conditions used to determine the nanoencapsulation efficiency. 

Volatile compounds present in the headspace were classified into eight olfactory families: citrus, fruity, floral, green, herbal, musk, oriental, and woody. The definitions available in The Good Scents Company Information System and Flavornet and human odor space databases were used as the criteria. The Odor Value (${OV}_{i}$) of each compound was calculated by Equation (3), using the concept of odor threshold (${ODT}_{i}$), which is the minimum gas concentration that can be perceived by the human nose. The Odor Value is a quantitative parameter that defines the intensity of a fragrant component as the ratio between its headspace concentration ($C_{i}^{g}$) and its odor threshold in the air, which is evaluated by panelists and is available in the literature [30].

(3) $${OV}_{i}=\frac{C_{i}^{g}}{{ODT}_{i}}$$

Since the literature definition of the classification of a pure component differs or assigns more than one olfactory family to the component, olfactory families can be considered as defined by Brechbill [31]; when other families are also referred to, they are used as secondary families, according to the order of relevance. A weight factor was used to explain the presence of nuance (Table 1). The Odor Value (${OV}_{j})$ for each olfactory family j was calculated.

This model considers that primary families are perceived more intensely than secondary or tertiary families. Thus, the Odor Values for each olfactory family were calculated using Equation (4):

(4) $${OV}_{j}=\sum_{i=1}^{N}w_{i}^{j}{OV}_{i}$$

where $w_{i}^{j}$ is the component factor for the family according to Table 1. Because perfumery radar diagrams are independent of total aroma intensity, the OVs of these olfactometric families are normalized according to Equation (5) [32]:

(5) $${OV^{\prime }}_{j}=\frac{{OV}_{j}}{\sum_{j=1}^{L}{OV}_{j}}$$

where ${OV^{\prime }}_{j}$ is the normalized Odor Value for the j family and L is the number of olfactory families defined. Through this transformation, it was possible to compare all perfumery radars on a scale, regardless of odor intensity, and plot them on a radar-like graph. Only compounds with expressive values were used for radar construction.

### 2.8. Air Diffusion

The Stefan tube was used for laboratory-scale aroma-diffusion and -release experiments [33], and was recently used for the same purposes by da Silva et al. [25]. A total of 5 mL of each formulation was placed in a glass container coupled to the bottom of a diffusion tube. For diffusion analysis of pure essential oils, 1 mL of each oil was used. The headspace samples (100 μL) at each distance from the source were collected with a gastight syringe and analyzed by GC-MS in the splitless, mode with all other parameters unchanged. Calibration curves were constructed from the standard headspace compositions of major essential oil compounds. For calibration, 1 mL samples of pure components were prepared in 20 mL vials. After 24 h to establish equilibrium, headspace samples were withdrawn by manual sampling. The data were collected in triplicate, maintaining a controlled temperature of 25 °C and varying the injection split from 5 to 400.

Mathematical modeling of the headspace data collected at the first sampling port (130 mm) was performed using the Peppas equation [34] (Equation (6)):

(6) $$\frac{M_{t}}{M_{\infty }}=kt^{n}$$

where *M_t_* is the total mass released in a time t and $M_{\infty }$ is the total mass for the release conducted for an infinitely large time.

This model presents two adjustable parameters: the constant k incorporates structural and geometric effects, and the kinetic order n is estimated by the least-squares method using the Nelder–Mead Simplex method [35], implemented in the MATLAB optimization toolbox. The literature on controlled release presents several mathematical models based on the different mechanisms that occur during release [36,37,38]. The Peppas model [39] was chosen because it is indicated when it is not known a priori which mechanism is dominant for the release case under analysis [40]. The values of the model parameters, when estimated from the adjustment of experimental data, will allow the identification of the mechanisms for release, in addition to representing the release kinetics curve. Due to the lack of knowledge about the release mechanism for the *Achyrocline satureioides* essential oil, the Peppas model was selected for this work.

## 3. Results and Discussion

### 3.1. Chemical Analysis

The total essential oil volume obtained by steam distillation was 5 mL, with a density of 845 ± 2 kg/m^3^. The *A. satureioides* essential oil had a light-yellow color, and the components identified by GC-MS are presented in Table 2. Unidentified compounds were omitted, corresponding to 6.82% of the total detected area. This composition corresponded to that reported in the literature for a pressure of 2.5 bar [7]. The essential oil had two major compounds: α-pinene (39.17%) and β-caryophyllene (18.71%). Under the same conditions, these compounds were previously found in proportions of 10.07% and 26.49%, respectively [7]. Four other compounds in relevant quantities were identified: α-humulene (4.79%), α-copaene (4.09%), δ-cadinene (4.05%), and β-selinene (3.01%). The remainder consisted of minor components, accounting for 22.37% of the essential oil. The composition of the essential oil is a function of factors such as the season in which the plant was collected, the characteristics of the soil, and the amount of sunlight on the plant [41,42,43]. In addition, the method of extraction, as well as its operational conditions, are determining factors in the composition of the essential oil [7,44]. In the case of marcela, the composition of the volatile oil is quite similar in qualitative terms, but different in quantitative terms, as can be seen in the available articles [7,23,45,46].

### 3.2. Characterization of the Nanocapsules

PCL nanocapsules containing *A. satureioides* essential oils were prepared using a nanoprecipitation method. The hydrodynamic diameters, as well as the polydispersity index and zeta potentials of the nanocapsules, are listed in Table 3.

The PDI values were between 0.137 and 0.249, indicating low dispersity. The values of the zeta potential were between −24.03 mV and −35.60 mV. Formulations M1 and M4 presented a higher level of dispersity, as the zeta potential deviations and their coefficients of variation were high; therefore, these samples were discarded. Formulation M3 was considered the most stable, with the smallest particle size (178.30 ± 1.18 nm) and the lowest PDI (0.137 ± 0.014); in addition, it exhibited a zeta potential below −30 mV (−31.03 ± 1.80 mV). These results agree with those reported by Ritter et al. [24] and Baldissera et al. [23], who found that the particles obtained using a vortex were smaller.

Nanoparticles with zeta potentials between −10 and +10 mV are considered relatively neutral, while nanoparticles with zeta potentials greater than +30 mV or less than −30 mV are considered strongly cationic and strongly anionic, respectively. As cell membranes are negatively charged, the zeta potential can affect the tendency of the nanoparticle to permeate membranes, with cationic particles generally showing greater toxicity associated with cell disruption [47].

Sample M3 presented a lower PDI and stronger anionic zeta potential, as well as lower CV, as respective properties (Table 3); therefore, it was selected as the best formulation to continue the tests.

Nanoemulsions with the same mass of oil were prepared and denoted as EM3. Formulations containing only the polymer without the addition of oil were also prepared and denoted PP. A comparison of M3, EM3, and PP is presented in Table 4.

Sample EM3 had a particle diameter almost twice that of M3, with a higher dispersity index. A high polydispersity index (>0.1) indicates low homogeneity in the sample size distribution.

**Figure 1:**
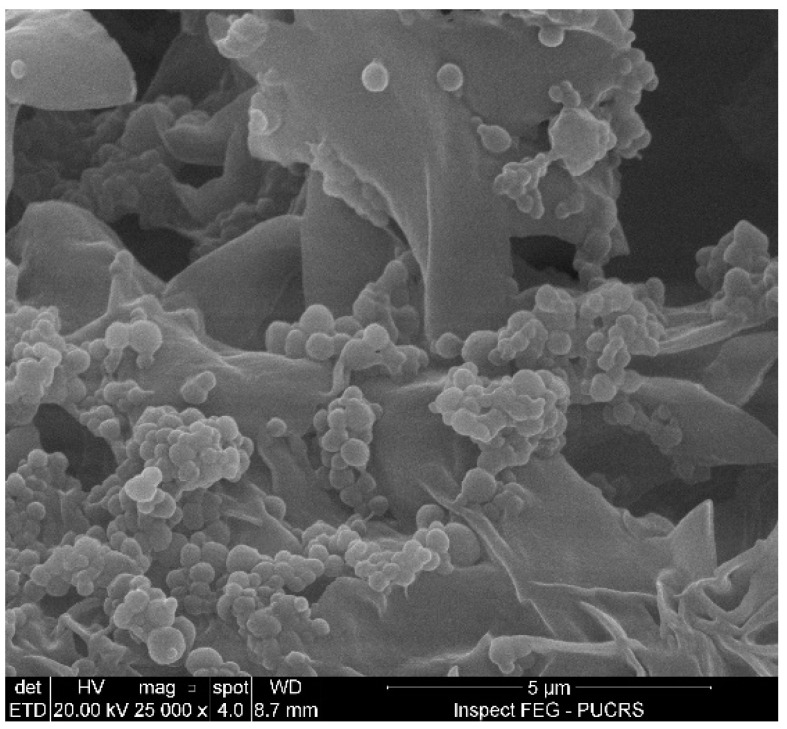
Particle morphology obtained by field-emission scanning electron microscopy (FEG-SEM).

The produced particles presented a spherical and homogeneous morphology, according to the FEG-SEM images shown in Figure 1.

**Figure 2:**
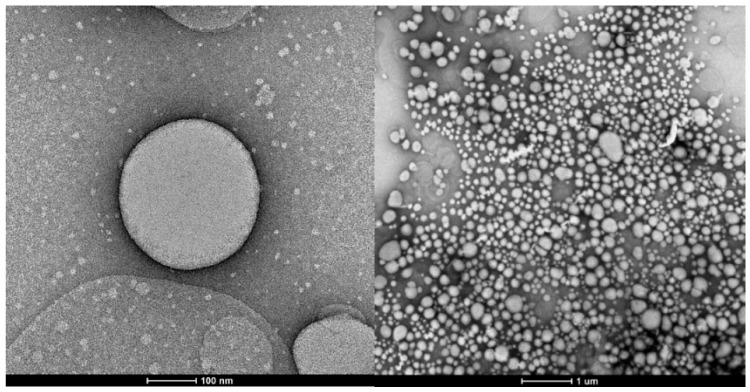
Particle morphology obtained by transmission electron microscopy (TEM) with an approximation of 100 nm (**left**) and 1 µm (**right**).

The morphology was confirmed by TEM, whose images are presented in Figure 2. It was possible to verify the spherical morphology of the particles and confirm the size data obtained via DLS. The particles presented a spherical and homogeneous morphology, as expected, because the preparation of the particles started from a nanoemulsion using a surfactant, where the spherical shape corresponds to the formation of micelles of this amphiphilic compound. Traditional nanoparticles produced using bottom-up techniques are limited to spheres, partly because of the lack of fabrication technology to control their shapes. These techniques rely on the self-assembly and aggregation of nanoparticles and depend on various factors, such as thermodynamic energy minima and entropy limitations, or factors affecting molecular self-assembly [48]. The energy-minimizing stable structures tended to be spherical, with the least surface per-unit volume. However, with the same volume, non-spherical particles have a larger surface area than spherical particles, giving rise to a larger flux per-unit volume [49]. The optimal morphology depends on the formulation goal, with the spherical shape better controlling the flux and preserving the essential oil, which is the case here.

The findings of this study indicate that the particle size of the nanostructures varies significantly, based on the essential oil used, as the composition of the volatile extract influences the particle size. For instance, using the same methodology with polycaprolactone to encapsulate essential oil from *Hedychium coronarium* [25], it was observed that the particles obtained with a polymer-to-*A. satureioides* essential oil mass ratio of 1:3 were approximately 10% larger than those derived from *H. coronarium*. This trend persisted even with a 20% higher concentration of essential oil when comparing the nanoemulsions.

### 3.3. Essential Oil Encapsulation Efficiency

Table 5 presents the remaining mass percentages at the end of the process with respect to the initial mass used for the formulation of the selected compounds. All oil retained in the nanoemulsion was successfully encapsulated because most of the compounds were not detected in the filtrate of the nanoparticle formulation. Therefore, the polymer protects the oil during rotary evaporation, indicating high encapsulation efficiency. 

Other compounds detected in nanoemulsion EM3 were β-pinene, limonene, p-cymene, and α-copaene. In the filtrate analysis of M3, the only detectable compound was β-caryophyllene. Therefore, it is assumed that all α-pinene that was not lost in the formulation process was involved with the biopolymer, in addition to the other non-quantified compounds.

**Figure 3:**
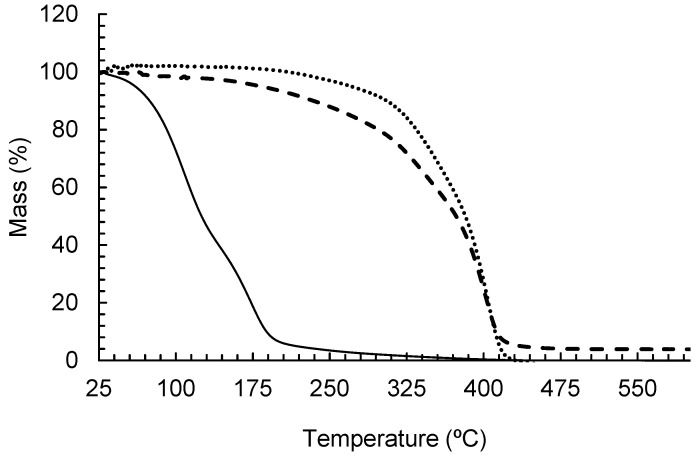
TGA curves for the *A. satureioides* essential oil nanocapsules (– – –), *A. satureioides* essential oil (—) and PCL particles (····).

The thermogravimetric analysis results are presented in Figure 3 as a mass loss-percentage curve versus temperature.

As expected, the PCL nanoparticles increased the thermal stability of the essential oils. The pure essential oil presented degradation in the range of 74 °C to 200 °C, while the nanocapsules presented degradation from 124 °C to 400 °C with a kinetic order close to zero. This demonstrates that the oil evaporation from inside the particles occurs in parallel with polymer degradation, which rapidly increased after 325 °C.

### 3.4. Results on Stability and Release

**Figure 4:**
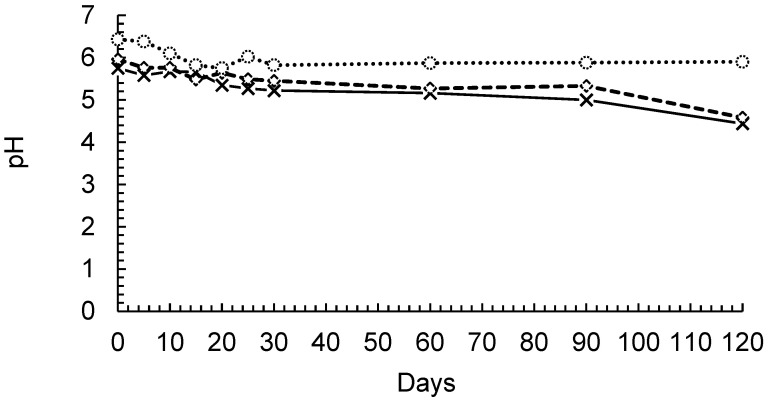
pH monitoring of nanocapsules (-◊-), nanoemulsions (-x-), and PCL particles (-○-).

The pH of the three evaluated formulations remained stable until the 90th day, when it decreased slightly, except for the PP solution, as shown in Figure 4. The nanocapsules, nanoemulsions, and PCL particles showed slightly acidic characteristics, which is consistent with previously reported data for formulations containing PCL and various drugs [50]. 

The maintenance of pH in the formulations also indicated that there was no significant polymer degradation, which has been previously reported for up to 8 months [51,52]. This suggested that the oil remained encapsulated because the nanoemulsions had slightly lower pH values than the nanocapsule formulations. The decrease in the pH value seems to be directly related to the nanoemulsion stability, as the formulations presented similar behaviors. Additionally, the polymer remained stable for even longer periods.

**Figure 5:**
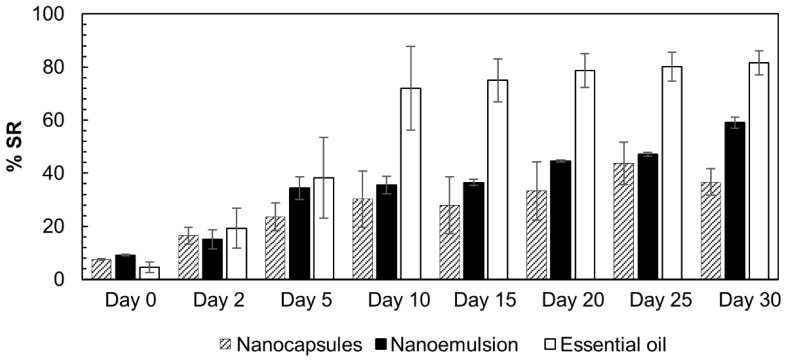
DPPH radical scavenging assay with the *A. satureioides* oil nanocapsule formulation (crosshatch), nanoemulsion (black), and free essential oil (white).

The antioxidant activity of the formulations presented in Figure 5 was evaluated to assess their stability and essential oil release/evaporation processes. On day zero, the absorbances of the formulations and free essential oil were measured after 2 h of reaction. The essential oil presented low antioxidant activity (%SR = 4.5), which confirmed the data reported in the literature [14].

Initially, the nanocapsules and nanoemulsions presented higher values than the free oil, possibly improving the availability of antioxidant compounds. From the 10th day, the scavenging rate of the free essential oil increased significantly, and reached its highest level (80%) after 20 d. From the 10th day, the M3 and EM3 formulations reached approximately 40% radical scavenging activity and maintained this level until the 25th day. On the 30th day, the nanocapsules continued to exhibit 40% SR, whereas that of the nanoemulsions increased to 60%. These results indicate that the presence of the nanoparticles promotes the controlled release of antioxidant compounds. The oil had essentially no additional activity after the 10th day. Therefore, the activity of the formulations was significantly superior to that of the DPPH solution.

### 3.5. Results from Perfumery Radar

The classification of the compounds present in the essential oil headspace within the eight olfactory families is shown in Table 6, along with the relevant properties for discussion. The presented values demonstrate that the fragrance intensities have considerable differences, mainly due to differences in the vapor pressure values, as this property has the greatest contribution to the headspace concentration. Therefore, the mixture behavior can be approximated based on the vapor pressure or volatility of the compounds (the usual methodology used for classifying the types of notes).

**Figure 6:**
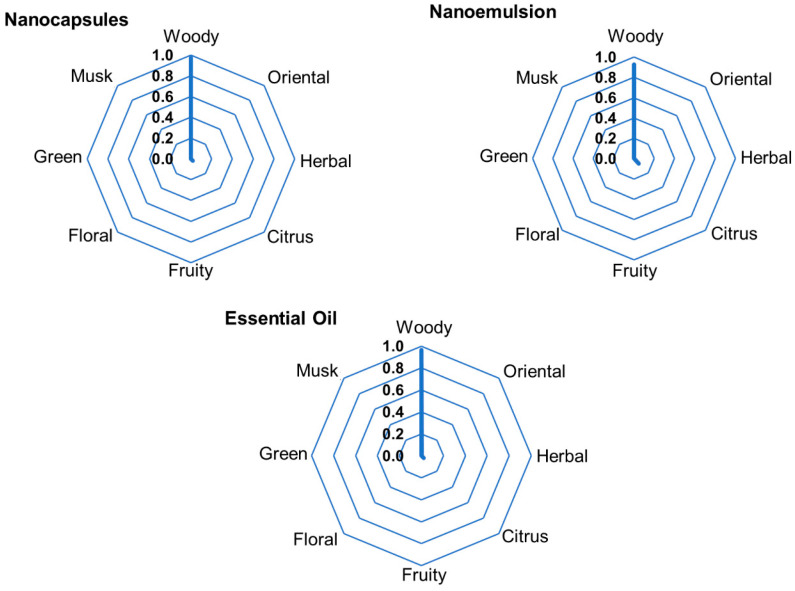
Experimental perfumery radars obtained from the headspace of nanoformulations containing *A. satureioides* essential oil and pure essential oil.

The perfumery radars (PRs) of the formulations containing *A. satureioides* essential oil are presented in Figure 6. Despite the close values in the vapor pressure between α-pinene, camphene, and β-pinene, their aromatic intensity values are considerably different, since their concentrations in the liquid phase are in an approximately 40:2:1 ratio. α-pinene, β-pinene, limonene, and β-caryophyllene were considered for the perfumery radar, since their headspace concentrations were significant; all other compounds presented in Table 7 were only present in trace amounts. There were no differences among the PRs of the emulsions, nanoparticles, and pure essential oils, because α-pinene is the dominant compound, which gives the *A. satureioides* essential oil its characteristic woody aroma.

### 3.6. Diffusion and Modeling

**Figure 7:**
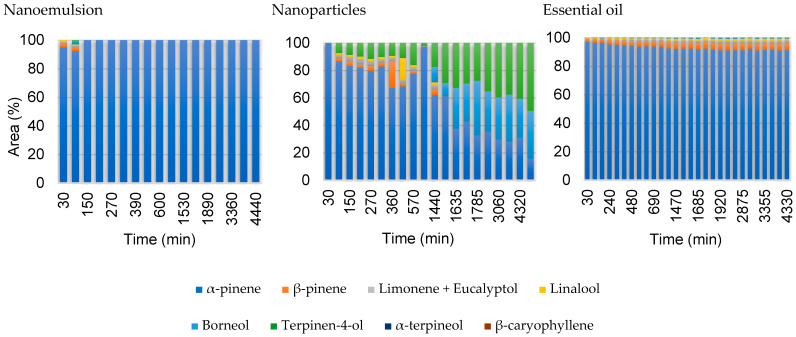
Comparison of the headspace composition in area percentage for the two formulations and the pure essential oil of *A. satureioides* collected at the first sampling port 130 mm (SP1).

The comparison between the composition profile of the formulations containing *A. satureioides* oil and the pure oil at port SP1 (130 mm from the source) is presented in Figure 7. It is possible to note that even for a distance close to the source, the composition of the vapor phase varies little over time in the case of the nanoemulsion, so the profiles for this formulation at distances greater than this are not presented.

**Figure 8:**
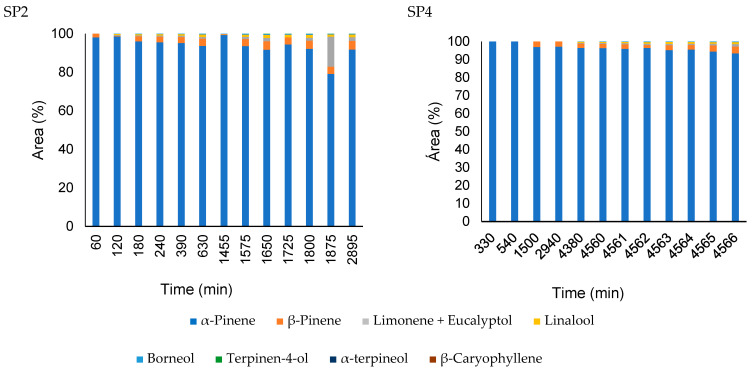
Comparison of the headspace composition of pure *A. satureioides* essential oil (EM3) in the SP2 (250 mm) and SP4 (1500 mm) ports.

**Figure 9:**
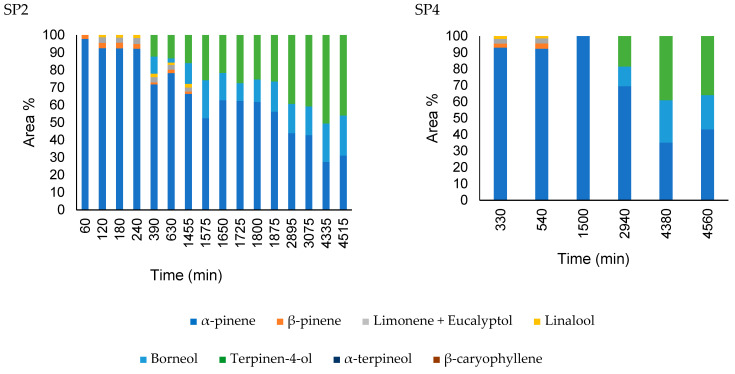
Comparison of the headspace composition of nanoparticles containing *A. satureioides* essential oil (M3) in the SP2 (250 mm) and SP4 (1500 mm) ports.

The headspace diffusion profiles for the nanocapsules and pure oil obtained in the second and fourth sampling ports, 250 and 1500 mm from the liquid–vapor interface respectively, are presented in Figure 8 and Figure 9, using the same layout as da Silva et al. [23]. The gas-phase composition of nanoemulsion containing *A. satureioides* essential oil (EM3) in the sampling ports SP2 and SP4 was constant, and only α-pinene was detected during the analysis, with traces of other compounds, which justifies the non-inclusion of these nanoemulsion graphs in Figure 8 and Figure 9. 

From the headspace composition profile, it is clear that the major compounds in the liquid phase of the pure essential oil diffusion are not present in the same proportions as those in the gas phase. As expected, this behavior was mainly due to the significant difference in the vapor pressure of the essential oil components, as well as their interactions in the liquid phase (non-idealities). For the *A. satureioides* oil, α-pinene, and β-caryophyllene, the major compounds had vapor pressures of 513 Pa and 4 Pa at room temperature, respectively.

The pure essential oil profile was constant over four days of analysis, even for minor compounds. α-pinene, with a molar mass of 136.23 g.gmol^−1^, diffused more quickly than α-copaene and β-caryophyllene, which are isomers with 204.35 g.gmol^−1^ molar mass. Low percentages of other compounds were observed in the initial minutes of diffusion. 

The gas-phase composition profile of the nanocapsule release was significantly different from that of the nanoemulsion. Initially, α-pinene dominated the composition, indicating that the free α-pinene in the solution initially evaporated in a greater proportion. After 90 min, the β-pinene composition increased to approximately 7%. α-copaene appeared after 1440 min of diffusion, reaching 30% of the composition in 1635 min. The nanoemulsion presented a profile similar to the diffusion profile of the essential oil; however, in this case, there was a greater predominance of α-pinene.

In general, diffusion through the polymer shell decreased with an increase in molecular size, decrease in vapor pressure, and increase in the partition coefficient of the aromas [53]. The molecular mass, conformation, chemical composition, and physical state of the coating material can influence its physical barrier properties. In contrast, for the highest level of polymerization, less aroma retention has been reported in the literature, probably because of the lower level of interaction between the aroma and carrier material [54].

The analyses revealed distinct compositions in the released gas phase (see Figure 7) from *A. satureioides* nanoparticles and nanoemulsions. In contrast, the nanostructures designed for the controlled release of *H. coronarium* essential oil showed similar gas-phase compositions, regardless of the specific nanostructure employed. This divergence highlights the varied interactions between the components of the essential oils and the nanostructures, emphasizing the importance of both the source material and the encapsulating medium in determining the characteristics of the final product.

The semi-empirical model proposed by Peppas [34] provides information on the type of mass transport that controls the release of the encapsulated substance into the medium. Table 7 presents the values of constant k, which incorporates structural and geometric effects, and exponent n, which indicates the type of physical phenomenon associated with release. The release profile, along with the Peppas model, is shown in Figure 9.

The geometric shape of the particles produced was spherical, as observed in Figure 2. For this geometry, according to Peppas [55], the Fickian diffusion mechanism is characterized by the value of n equal to 0.43 for the release model. However, the values of n found for the release tests of the formulated nanostructures indicate that the release is occurring by non-Fickian mechanisms (anomalous transport), or even that the diffusion is occurring simultaneously, together with other release mechanisms.

**Figure 10:**
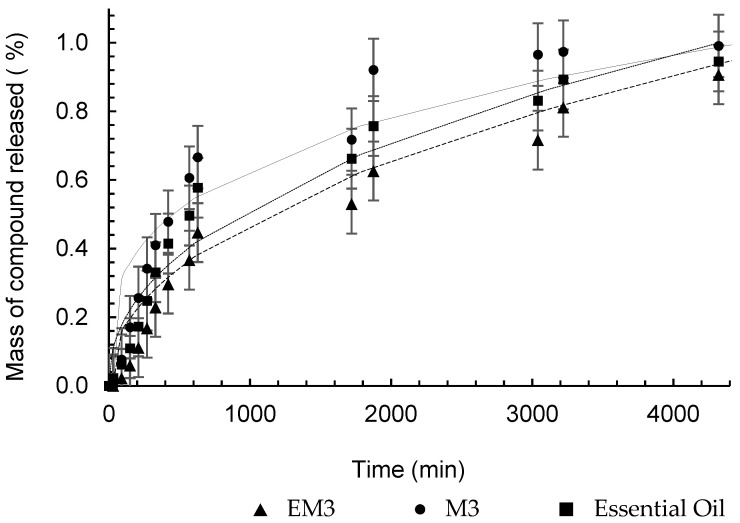
Peppas analytical model fitted to the experimental release data of α-pinene from nanocapsules (—), nanoemulsions (– – –), and pure essential oil (····).

**Figure 11:**
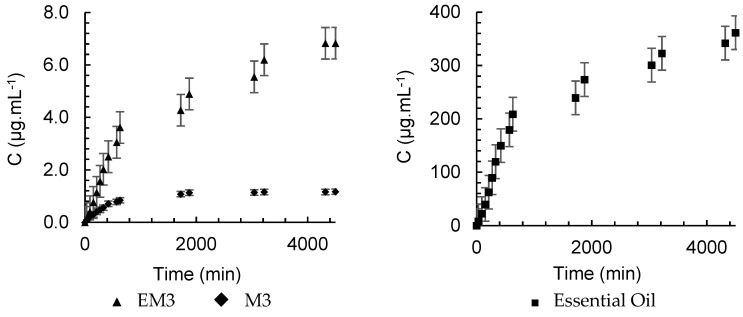
Headspace concentration profile for α-pinene in nanoemulsions and nanoparticles containing *A. satureioides* oil and pure oil in SP1.

The release kinetics of α-pinene from M3, presented in Figure 10, was faster than that of EM3, which is evidenced by the values of k, which were 0.0786 min^−n^ and 0.0189 min^−n^, respectively. This result appears to contradict the purpose of encapsulation, but by analyzing the concentration versus time, shown in Figure 11, the difference in the amount of α-pinene released is evident. The polymer accelerated the release kinetics, since the stationary period was reached at 630 min, but it also retained more α-pinene, which promoted its controlled release in low doses for a longer period. The kinetic behavior of pure oil showed no significant difference between the formulations and the essential oil (Figure 11), but the difference between the concentration of the compound released at every instant was relevant. The maximum concentration of α-pinene in the nanoparticle gaseous phase was 1.16 μg.mL^−1^, whereas in the nanoemulsion, it was no more than 6.83 μg.mL^−1^. For the pure oil, after 30 min of diffusion, the concentration in SP1 was already 8.26 μg.mL^−1^ and the maximum measurement was 361.5 μg.mL^−1^.

The release kinetics depend on different properties of the encapsulating polymers, including molecular weight, degree of polymerization, and thermal stability. There are reports indicating a decrease in the release rate with the use of high-molecular-weight polymers [56,57]. However, for the highest degree of polymerization, a lower aroma retention is reported in the literature, probably due to the lower degree of interactions between the aroma and the carrier material [54]. Another important aspect of the polymer in encapsulation is its thermal stability; polycaprolactone has high thermal stability [58]. Its degradation occurs at temperatures well above the volatilization and degradation temperatures of the major components of *A. satureioides*, α-pinene and β-caryophillene.

Release studies of formulations aimed at protecting flavor and fragrance have been performed using pure compounds [59,60,61]. The study of evaporation and air diffusion of compounds in a complex mixture such as essential oils is challenging, and the adopted methodology, through marker compounds, is pioneering and presents consistent results, in addition to those presented by da Silva et al. [25].

One of the reasons for increasing the release time is to artificially reproduce the behavior of aromatic plants, which is the slow and permanent release of secondary metabolites associated with the protection of the plant species. In the case of essential oil encapsulation, the objective is to increase stability and control their release [62], in addition to increasing the release time of aromatic substances in applications such as perfumery and cosmetics. This increase in bioavailability is revealed by the permanence of the antioxidant power. 

## 4. Conclusions

The characterization of the essential oil obtained by steam distillation of the *Achyrocline satureioides* inflorescences was carried out in GC-MS, and the major compounds were identified as α-pinene (39.17%) and β-caryophyllene (18.71%). Poly-ε-caprolactone nanocapsules containing the essential oil were prepared to provide a physical coating for the terpenic compounds of the oil, which aims to prolong the time of these compounds in solution and generate a controlled release. Dynamic light scattering analysis revealed that the nanoparticles prepared in the proportion of 1:3 (polymer/oil) presented a lower polydispersity index, particle size, and zeta potential; therefore, it was considered the best formulation. The stability of the formulations was evaluated by their pH, which indicates a minimum 120-day period of viability. Both nanoemulsions and nanoparticles promote a controlled release of the compounds, resulting in an extended antioxidant activity.

The evaluation of the diffusion profile also indicates the controlled release of the encapsulated and nanoemulsion formulations regarding their concentration levels compared to the essential oil. Physicochemical parameters involved in the release step were obtained by mathematical modeling using the Peppas model. The polymer accelerated the release kinetics, but it also retained more α-pinene, which promoted its controlled release for a longer period. The difference between the concentration of the compound released at every instant was quite relevant. A maximum concentration of 1.16 μg.mL^−1^ of α-pinene in the gas phase of the nanocapsules and 6.83 μg.mL^−1^ in the nanoemulsion were observed, while for pure oil the maximum measure was 361.5 μg. mL^−1^.

## Figures and Tables

**Table 1:** Weight distribution ($w_{i}$) for each olfactory family.

Number of Families	Family		
	Primary	Secondary	Tertiary
1	100%	-	-
2	70%	30%	-
3	60%	30%	10%

**Table 2:** GC-MS analysis of *A. satureioides* essential oil extracted by steam distillation at 2.5 bar.

Compound	RI_c_^a^	RI_t_^b^	Area (%) ^c^		
α-Pinene	930	932	39.17	±	0.67
Camphene	941	946	1.81	±	0.07
β-Pinene	969	974	1.05	±	0.16
p-Cymene	1020	1022	0.39	±	0.07
Limonene	1024	1024	2.73	±	0.12
Eucalyptol	1026	1026	0.23	±	0.01
Z-β-Ocimene	1036	1032	1.76	±	1.54
E-β-Ocimene	1046	1044	0.15	±	0.01
γ-Terpinene	1054	1054	0.24	±	0.01
Terpinolene	1083	1086	0.51	±	0.11
Exo-fenchol	1108	1118	0.32	±	0.18
Terpinen-4-ol	1160	1174	0.66	±	2.20
α-Terpineol	1186	1186	0.37	±	0.20
α-Copaene	1369	1374	4.09	±	0.10
Isocaryophyllene	1399	1408	0.23	±	0.03
β-Caryophyllene	1414	1417	18.71	±	3.00
β-Gurjunene	1431	1431	0.78	±	0.38
α-Guaiene	1443	1437	0.26	±	0.27
α-Humulene	1447	1452	4.79	±	2.08
Alloaromandrene	1453	1458	0.60	±	0.46
γ-Muurolene	1470	1478	1.47	±	0.35
β-Selinene	1479	1489	3.01	±	0.05
α-Muurolene	1494	1500	1.07	±	0.10
γ-Cadinene	1507	1513	1.40	±	0.11
δ-Cadinene	1518	1522	4.05	±	2.82
trans-Cadina-1.4-diene	1526	1533	0.33	±	0.20
α-Cadinene	1531	1537	0.29	±	0.29
α-Calacorene	1536	1544	0.78	±	0.44
Caryophyllenyl alcohol	1563	1570	1.06	±	0.40
epi-α-Cadinol	1635	1638	0.61	±	0.22
α-Cadinol	1649	1652	0.26	±	0.13
Total identified			93.18		0.54

^a^ Calculated retention index (RI_c_) regarding a series of alkanes (C_8_–C_20_) in a DB-5 column (30 m × 0.25 mm × 0.25 μm). ^b^ Theoretical retention index (RI_t_) in the HP-5MS series [21]. ^c^ Percentage area of each peak in the total chromatogram. Values are the averages of triplicate measurements without a response factor ± standard deviation.

**Table 3:** Results of dynamic light scattering analysis (DLS) for the nanocapsules containing *A. satureioides* essential oil.

Formulation	Particle Size (nm)	CV (%)	PDI	CV (%)	ZP (mV)	CV (%)
M1	168.77 ± 0.40 ^a^	0.24	0.146 ± 0.011 ^a^	7.534	−29.40 ± 6.60 ^a^	22.45
M2	174.10 ± 0.82 ^a. b^	0.47	0.147 ± 0.020 ^a^	13.605	−24.03 ± 1.93 ^a^	8.03
M3	178.30 ± 1.18 ^b. c^	0.66	0.137 ± 0.014 ^a^	10.219	−31.03 ± 1.80 ^a^	5.80
M4	181.20 ± 2.79 ^c^	1.54	0.249 ± 0.007 ^b^	2.811	−35.60 ± 10.67 ^a^	29.97
M5	190.83 ± 4.41 ^d^	2.31	0.228 ± 0.007 ^b^	3.070	−32.87 ± 0.85 ^a^	2.59

Values with equal superscripts are not significantly different (*p* < 0.05).

**Table 4:** DLS analysis for the best nanoparticle formulation (M3), corresponding nanoemulsion (EM3), and polymer particles without *A. satureioides* essential oil (PP).

Formulation	Particle Size (nm)	CV (%)	PDI	CV (%)	ZP (mV)	CV (%)
M3	178.30 ± 1.18 ^a^	1	0.137 ± 0.014 ^a^	11	−31.03 ± 1.80 ^a^	6
EM3	329.67 ± 56.8 ^b^	17	0.401 ± 0.056 ^c^	14	−31.87 ± 1.30 ^a^	4
PP	187.50 ± 1.25 ^a^	1	0.243 ± 0.008 ^b^	3	−28.07 ± 2.01 ^a^	7

Values with equal superscript values indicate no significant difference (*p* < 0.05).

**Table 5:** Efficiency in the *A. satureioides* essential oil encapsulation process in PCL nanocapsules.

Formulation	Compound	% Mass in Emulsion	% In Filtrate	Encapsulation Efficiency (%)
M3/EM3	α-Pinene	72	-	≥72
	β-Caryophyllene	9	trace	9 < % < 100

**Table 6:** Relevant parameters for the *A. satureioides* headspace perfumery radar.

Compound	Molecular Formula	$M_{i}$ (g.gmol^−1^)	$P^{vap}$ (Pa)	ODT ^a^ (mg.m^−^³)	ρ ^b,c^ (kg.m^−^³)	Odor Family ^d^
α-Pinene	C_10_H_16_	136.23	513	0.240	860	Woody (pine)
Camphene	C_10_H_16_	136.23	450	-	839	Woody (camphor)
β-Pinene	C_10_H_16_	136.23	390	0.180	873	Woody (pine), oriental
Limonene	C_10_H_16_	136.23	264	0.619	854	Citrus
Z-β-ocimene	C_10_H_14_	134.22	208	0.010	810	Floral, herbal
α-Copaene	C_15_H_24_	204.35	5	-	939	Woody, oriental
β-Caryophyllene	C_15_H_24_	204.35	4	1.500	907	Woody, oriental
α-Humulene	C_15_H_24_	204.35	1.1	-	889	Woody
β-Selinene	C_15_H_24_	204.35	2.3	-	914	Herbal
δ-Cadinene	C_15_H_24_	204.35	0.9	-	910	Herbal, woody

^a^ van Gemert [30]; ^b^ PubChem (https://pubchem.ncbi.nlm.nih.gov/) accessed on 1 March 2024; ^c^ ChemSpider (http://www.chemspider.com/) accessed on 1 February 2024; ^d^ Descriptions are available in the Flavornet database (http://www.flavornet.org) accessed on 1 March 2024 and The Good Scents Company information (http://www.thegoodscentscompany.com/) accessed on 1 June 2024.

**Table 7:** Parameters from the modeling of the experimental data using Peppas equation for the *Achyrocline satureioides* oil nanocapsules, nanoemulsions, and pure essential oil.

α-Pinene	n	k (min^−n^)	R²	R² adj	SQE	RMEQ
EM3	0.47	0.0189	0.9699	0.9900	0.0453	0.0549
M3	0.30	0.0786	0.9273	0.9758	0.0827	0.0742
Essential oil	0.45	0.0234	0.9461	0.9820	0.0876	0.0764

## Data Availability

Data are contained within the article.
